# Black Garlic Improves Heart Function in Patients With Coronary Heart Disease by Improving Circulating Antioxidant Levels

**DOI:** 10.3389/fphys.2018.01435

**Published:** 2018-11-01

**Authors:** Jingbo Liu, Guangwei Zhang, Xiaoqiang Cong, Chengfei Wen

**Affiliations:** Department of Cardiovascular, The First Hospital of Jilin University, Changchun, China

**Keywords:** coronary heart disease, congestive heart failure, quality of life, left-ventricular ejection fraction, black garlic

## Abstract

**Background:** Black garlic (BG) has many health-promoting properties.

**Objectives:** We aimed to explore the clinical effects of BG on chronic heart failure (CHF) in patients with coronary heart disease (CHD).

**Design:** The main components of BG were measured by gas chromatography–mass spectrometry (GC–MS) and its antioxidant properties were determined by the clearance rate of free radicals. One hundred twenty CHF patients caused by CHD were randomly and evenly assigned into BG group and placebo group (CG). The duration of treatment was 6 months. Cardiac function was measured according to the New York Heart Association (NYHA) functional classification system. The following parameters were measured, including walking distance, BNP precursor N-terminal (Nt-proBNP), left-ventricular ejection fraction (LVEF) value, and the scores of quality of life (QOL). The circulating antioxidant levels were compared between two groups.

**Results:** There are 27 main compounds in BG with strong antioxidant properties. BG treatment improved cardiac function when compared with controls (*P* < 0.05). The QOL scores and LVEF values were higher in the BG group than in the CG group while the concentration of Nt-proBNP was lower in the BG group than in the CG group (*P* < 0.05). Circulating antioxidant levels were higher in the BG group than in the CG group. Antioxidant levels had positive relation with QOL and LVEF values, and negative relation with Nt-proBNP values.

**Conclusion:** BG improves the QOL, Nt-proBNP, and LVEF in CHF patient with CHD by increasing antioxidant levels.

## Introduction

Chronic heart failure (CHF) may be caused by myocardial abnormalities, which result in systolic and/or diastolic ventricular dysfunction, abnormalities of the valves, pericardium, endocardium, heart rhythm, a reduced cardiac output, or brain abnormalities (12). Vasomotor function cannot meet the needs of systemic metabolism, resulting in hemodynamic abnormalities and neurohormonal activation (Hammadah et al., 2017). There are 26 million CHF patients worldwide and the prevalence of CHF is still increasing with population aging (Schmid et al., 2017). CHF is a common cause of death in the elderly (Andres et al., 2018; Clark, 2018). Five-year mortality rate of CHF is more than 20%, and seriously threatens human life (Nakajima et al., 2014). Considering its poor prognosis, it is critical to prevent the occurrence and development of CHF and to promote early rehabilitation of CHF patients.

Garlic is a kind of valuable atherosclerosis-preventing functional food (Alali et al., 2017). Many reports showed that garlic had lipid-lowering, plasma anticoagulant and antioxidant activities, and improves endothelial injuries (Gorinstein et al., 2007). The extract of garlic was effective to reduce blood pressure, arterial stiffness, inflammation, and other cardiovascular diseases (Ried et al., 2016). Garlic is a kind of feasible and promising functional food for individuals with cardiovascular disease (Aslani et al., 2016; Siddiqui et al., 2017).

**FIGURE 1:**
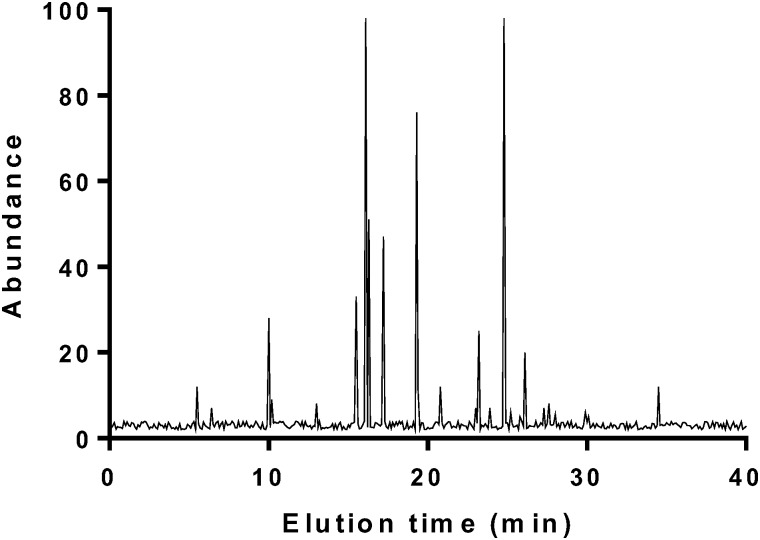
GC analysis of main components of BG.

Black garlic (BG) is a kind of deep-processed food made of fresh garlic under high temperatures and humidity. It can improve immune activity with fewer side effects (Nakasone et al., 2016). BG has many health-promoting properties: BG prevented the growth and induced apoptosis of HT29 colon cancer cells via phosphatidylinositol 3-kinase (PI3K)/Akt pathway, suggesting that BG may be effective in the therapy of colon cancer (9); BG had potential beneficial effects in the treatment of diabetes by increasing in the numbers of monocytes and granulocytes, and decreasing lymphocyte proliferation (10); BG has anti-allergic actions and may be beneficial as functional food in the prevention of allergic disorders (11). BG has various biological functions, including antioxidant (Lu et al., 2017; Sun and Wang, 2018), anti-inflammatory (Jeong et al., 2016), anticancer (Dong et al., 2014), antidiabetic (Abel-Salam, 2012), anti-allergic action (Yoo et al., 2014), and the improvement of lipid metabolism (Ha et al., 2015), cardiac (Czompa et al., 2018), and hepatic protection (Kim et al., 2011; Figure 1). However, the effects of BG on the CHF patients and the related molecular mechanisms remain unknown.

Chronic heart failure is a complex, and dynamic development process. Neuro-hormones, inflammation, and cytokines play an important role in this process. A large number of studies have shown that brain natriuretic peptide (BNP) is involved in the pathophysiological process of the development of CHF (Du et al., 2012). BNP is a neuro-hormone secreted by ventricular myocytes. Under normal circumstances, there is little BNP in the atria and ventricle. In many pathological conditions, such as the ventricular volumetric load and pressure overload, BNP concentration will be increased in the blood (Kou et al., 2016). The higher severity of heart failure will result in higher concentration of BNP (Hahn et al., 2016; Feng et al., 2017). BNP shows good specificity in differential diagnosis of CHF (Jin et al., 2018), cardiogenic dyspnea (Golshani et al., 2016), and lung-derived respiratory distress (Sun et al., 2015).

After being stimulated by cardiomyocytes, BNP is cleaved by proteases into Nt-proBNP and active BNP. The clinical application of Nt-proBNP and bioactive hormone BNP to CHF is similar. However, BNP has a 20 min half-life and is poorly stable *in vitro*. Comparatively, the half-life of Nt-proBNP is 60–120 min and stable *in vitro* (Mukherji et al., 2017). Therefore, the quantitative detection of plasma Nt-proBNP is more feasible. In this study, we explored the effects of BG on CHF patients with coronary heart disease (CHD). The improvement of quality of life (QOL) of CHF patients was compared with controls and the levels of Nt-proBNP were measured.

## Materials and Methods

### Measurement of the Component of Black Garlic

Raw garlic and BG were purchased from Shandong Sanjin Black Garlic Industry Co., Ltd. (Jinxiang, China). The difference for the main components between raw garlic and BG was listed in Table 1. BG was prepared from fresh garlic via the fermentation (60–80°C, 70–95% relative humidity) for 50 d. Thirty grams of raw garlic and BG was weighed, respectively, ground by a mortar, and placed in a 1000-mL round-bottomed flask. Thirty milliliters of sodium chloride and distilled water was added, and heated by temperature-controlled electric heating apparatus. After distillation, it was concentrated to 1.0 mL and transferred to a chromatography flask for gas chromatography–mass spectrometry (GC–MS) analysis.

**Table 1:** Comparison for the ingredients between raw garlic and BG (100 g).

Ingredients	Raw garlic	BG
Moisture (g)	65.31 ± 1.75	37.12 ± 3.70
Total sugar (g)	27.23 ± 0.52	48.47 ± 1.75
Reducing sugar (g)	0.47 ± 0.03	39.49 ± 0.74
Protein (g)	5.32 ± 0.08	10.26 ± 0.76
Crude fat (g)	0.35 ± 0.06	0.16 ± 0.05
Acid value (g)	0.47 ± 0.06	2.13 ± 0.14
5-HMF (mg)	0	8.732 ± 0.17
Total phenols (mg)	184.35 ± 14.12	482.46 ± 20.04
**Amino acid (mg)**		
Aspartic acid	14.66 ± 0.81	30.81 ± 1.43
Glutamate	60.5 ± 0.27	38.52 ± 1.14
Serine	75.06 ± 0.5	41.28 ± 0.69
Glycine	102.58 ± 10.36	13.17 ± 0.75
Histidine	73.42 ± 1.07	14.8 ± 0.21
Threonine	131.47 ± 0.68	36.71 ± 0.82
Arginine	1079.88 ± 1.05	845.16 ± 20.29
Alanine	27.3 ± 1.02	108.59 ± 11.05
Valine	41.71 ± 0.39	13.84 ± 0.72
Tyrosine	96.68 ± 8.45	90.19 ± 10.67
Valine	40.89 ± 2.25	56.72 ± 0.33
Methionine	491.38 ± 78.84	3.48 ± 1.01
Cysteine	3.75 ± 0.27	18.08 ± 0.79
Isoleucine	14.08 ± 1.03	21.93 ± 0.29
Leucine	9.2 ± 0.8	28.72 ± 0.85
Phenylalanine	30.55 ± 0.16	62.86 ± 1.73
Tryptophan	23.51 ± 0.67	7.65 ± 0.54
Lysine	120.39 ± 13.45	61.83 ± 0.27
Total	1943.77 ± 161.22	1486.65 ± 112.62

Agilent 7890 Gas Chromatograph and Agilent 7890 GC/5975CMS GC/MS were purchased from Agilent (Foster City, CA, United States). The following chromatographic conditions were used: Column: HP-5MS (60 m × 0.25 mm × 0.25 μm); inlet temperature, 250°C; injection quantity, 10 μL; split ratio, 4:1; carrier gas, He, 1.0 mL/min; temperature program, 50 (2 min) and 220°C (30 min) and heating rate, 4°C/min. The following mass spectrometry conditions were used: transmission line temperature, 240°C; EI source electron energy, 70 eV; electron multiplier voltage, 1635 V; mass scanning range, 0–450 amu; ion source temperature, 230°C; and quadrupole temperature, 150°C. GC–MS results were analyzed manually and compared with standard mass spectra to determine the chemical structure of each separated components.

### The Measurement of Antioxidant Properties of Garlic

#### Preparation of Extracts of Black Garlic

The fresh raw garlic and BG were dried to a constant mass and pulverized. One gram of raw garlic and BG sample mechanical powder, and 20 mL of a 50% ethanol solution were added at a ratio of 1:20 to the stock solution. The main components were extracted via ultrasonic at 30°C for 30 min, centrifuged, filtered, and diluted with 50% ethanol to 20 mL as a sample solution.

#### Determination of Antioxidant Capacity of Black Garlic

One hundred microliters of raw garlic and BG extracts was added to the 96-well transparent plates with different concentrations (0.25, 0.50, 1.00, 2.00, 4.00, and 8.00 mg/mL) and 100 μL of 0.2 mM 2,2-diphenyl-1-picrylhydrazyl (DPPH) solution was added. With the same method, the different concentrations of DPPH and methanol were mixed as a blank group, and solution and methanol were mixed as a control group. After being kept at room temperature for 30 min in the dark, the absorbance was measured at 517 nm and repeated three times. DPPH clearance = (1-(*D*1-*D*2)/*D*3) × 100%, where *D*1 was the sum of the absorbance of the DPPH solution and the sample solution; *D*2 was the sum of the absorbance of the sample solution and the extraction solvent; *D*3 was the sum of the absorbance of the DPPH solution and the extraction solvent.

#### Determination of the Reducibility of Fe^3+^

Two hundred microliters of raw garlic and BG extract (0.625, 1.250, 2.500, 5.000, and 10.000 mg/mL) was placed in 5-mL centrifuge tubes, respectively, and 0. 5-mL 0.2 mol/L phosphate-buffered saline (PBS), pH 6.6, and 0.5-mL 1% potassium ferricyanide solution were added. After being mixed, bath at 50°C for 20 min, and then 0.5 mL 10% ferric chloride solution (TCA) was added and centrifuged at 5000 r/min for 5 min. 0.5-mL supernatant was taken, and 0.5-mL distilled water and 0.1-mL 0.1% ferric chloride solution were added. The absorbance values were measured at 700 nm and repeated three times.

#### Clearance Rate of 2,2′-Azino-bis(3-ethylbenzothiazoline)-6-sulfonic acid (ABTS)-Free Radicals

Seven millimolar ABTS solution was prepared with PBS (pH = 7.4), and 7 mM ABTS and 2.45 mM potassium phosphate were mixed in equal volume. The solution was diluted with PBS until the absorbance reached 0.66 ± 0.03 at 734 nm. Twenty microliters of different concentrations of raw garlic and BG extracts (0.25, 0.50, 1.00, 2.00, 4.00, and 8.00 mg/mL) was added to the 96-well plate, and 150 μL of 0.2 mmol/ABTS stock solution was used as a control. After the reaction was performed at room temperature for 10 min, and the absorbance was measured at 517 nm. ABTS clearance rate = (*D*0-*D*)/*D*0 × 100%, where *D*0 was the sum of absorbance of ABTS working solution and PBS; *D* was the sum of absorbance of ABTS working solution and sample solution.

#### Determination of Oxygen Radical Absorption Capacity (ORAC)

With different concentrations of raw garlic, BG extract (0.25, 0.50, 1.00, 2.00, 4.00, and 8.00 mg/mL) and 20 μL Trolox standard (diluted with 75 mmol/L PBS, pH = 7.4) was added to a 96-well plate, incubated at 37°C for 20 min, and then 20 μL of 119 mM 2,2-azo-bis(2-amidino-propa) hydrochloride (ABAP) solution was added. The fluorescence intensity was measured with an excitation wavelength of 485 nm and an emission wavelength of 535 nm. The measurement time interval was 5 min and 35 measurements were continuously performed. The ORAC values were expressed in Trolox equivalents in mmol TE/gDW (Trolox equivalent per gram of dry weight).

### Participants

All procedures were approved by the human research ethical committee of Jilin University (Changchun, China) (Approval No. 20160713F). This trial was registered at http://www.chictr.org.cn/searchprojen.aspx, Clinical number: ChiCTR1800017999^1^. All patients agreed with consent form and signed their names. CHF patients were determined according to Framingham or modified Boston criteria for heart failure (Remes et al., 1992). The total scores of CHF were 8. Cardiac function was measured according to the New York Heart Association (NYHA) (Bredy et al., 2018). Grade I – the patients suffered from heart disease and physical activity was not limited. General physical activity would not cause fatigue, palpitations, dyspnea, or angina; Grade II – the patients suffered from heart disease, and physical activity was slightly limited. General physical activity could cause fatigue, heart palpitations, difficulty in breathing, or angina; Grade III – the patients had heart disease, and physical activity was significantly limited. Light physical labor could cause fatigue, heart palpitations, difficulty in breathing, or angina; Grade IV – the patients suffered from heart disease, and physical activity was completely lost. There were the symptoms of heart failure or angina during rest. Any physical activity could make the symptoms worse.

### Inclusion Criteria

At the same time Framingham’s heart failure qualitative diagnostic criteria and Boston heart failure quantitative diagnostic criteria; age 35–75 years; CHD was measured by using Doppler echocardiography (GE, Fairfield, CT, United States); left ventricular ejection fraction (LVEF) ≤ 50; heart function grades II–III.

### Exclusion Criteria

The participants would be excluded if they had a history of knee surgery within past 3 months, a systemic arthritic condition, and any other muscular, joint, or neurological condition affecting lower limb function. The patients had acute coronary syndrome, severe valvular heart disease, combined shock, methicillin-resistant *Staphylococcus aureus* (MRSA) infections, planned extra-cardiac surgery, combined hepatorenal, and other systemic diseases.

### Patients Grouping

From May 1, 2016 to June 30, 2017, a total of 489 CHF patients were screened. CHF patients caused by CHD were selected and evenly assigned into BG (received 20 g garlic daily) and placebo (CG) groups. Primary endpoints were based on the 1-month observation after randomization. The first primary endpoint consisted of mortality, stroke, and myocardial infarction to define the sample size 120. The duration of treatment was 6 months. The following parameters were measured: 6-min walking distance, BNP, EF value, scores of QOL, and blood lipid profiles. QOL was assessed by using the Minnesota Living with Heart Failure Questionnaire (MLHFQ) (Mogle et al., 2017). Routine treatment included oxygen inhalation, angiotensin-converting enzyme inhibitors (ACE-in), angiotensin receptor blockers (ARBS), beta-blockers, digitalis preparations (except digitalis contraindications), intermittent application of diuretics, cardiac resynchronization therapy (CRT), and implantable cardioverter defibrillator (ICD). All the CHF patients received a 30-min walking exercise for 5 days a week on a flat surface at their comfortable speed.

### Lipid Profile Analysis

Triglycerides (TG), total cholesterol (TC), low-density lipoprotein cholesterol (LDL-C), and high-density lipoprotein cholesterol (HDL-C) is associated with CHF risk or progression (Jin et al., 2018). Serum TG was measured by using an immunometric assay (Immulite 2000 Thyroglobulin, Los Angeles, CA, United States). Serum TC, LDL-C, and HDL-C were analyzed by using an automated chemistry analyzer (Olympus, Japan).

### Measurement of Circulating Antioxidant Levels

Five milliliters of blood was obtained from each patient. Circulating antioxidant levels were investigated by measuring the levels of malondialdehyde (MDA), and nitric oxide (NO), glutathione peroxidase (GSH-Px), and superoxide dismutase (SOD) via ELISA kits (Beyotime Institute of Biotechnology, Beijing, China).

### Clinical Examination of Therapeutic Results

Cardiac function was measured according to the functional classification system of the NYHA. Significantly effective: heart function was improved by two levels or more; Valid: heart function was improved by one level but less than two levels; Invalid: heart function was improved less than one level; Deterioration: heart function was reduced at one level or above. Total efficiency = (significant + effective)/total number of cases × 100%. Six minutes walking test was used to compare the patient’s walk distance before and after garlic consumption between the two groups. LVEF was measured by radionuclide ventriculography with patients in the supine position (Naar et al., 2014).

Blood urea nitrogen (BUN) was measured on an automatic biochemistry analyzer (Beckman Coulter LX20, Beckman, CA, United States) by using a BUN kit (Beckman Coulter, Inc., Brea, CA, United States). Serum was isolated from blood sample via centrifugation. Serum creatinine was measured by a creatinine kit (Biovision, Milpitas, CA, United States). BUN and creatinine were assessed at before and after BG therapy.

### Detection of BNP Precursor N-Terminal (Nt-proBNP)

Two milliliters of venous blood was collected in a standard tube containing an anticoagulant from all patients with an empty stomach. Roche Elecsys Nt-proBNP kit (Roche, Indianapolis, IN, United States) was used to measure the level of Nt-proBNP by using electrochemiluminescence immunoassay on Elecsys 1010/2010/modular analytics E170 immunoassay system (Roche Diagnostics GmbH, Mannheim, Germany).

### Statistical Analysis

All data were statistically analyzed by using SPSS20.0 statistical software. The measurement data were first tested for normal distribution and homogeneity of variance. If there was a normal distribution and homogeneity of variance, paired *t*-tests were used before and after treatment between the two groups. *T*-test was used for the comparison between the two groups, and those that did not meet the homogeneity of variance. Count data were examined using the χ^2^-test. Continuous data were ranked using the rank sum test. *P* < 0.05 was considered statistically significant.

## Results

### The Main Components of Black Garlic

There are 27 kinds of volatile components in fermented BG (Figure 1 and Table 2). Among them, higher volatile components are 3-vinyl 3,4-dihydro-1,2-dithiane and diallyl compounds, such as disulfide, 2-ethyltetrahydrothiophene, 2-vinyl-1,3-dithiane, *N*,*N*″-dimethyl thiourea, etc. BG has a high content of 2-ethyl tetrahydrothiophene, which gives fragrance.

**Table 2:** The main component of polyphenols of BG.

Components	Molecular formula	Molecular weight	Retention time (min)	Percent components
Allyl alcohol	C3H6O	58.08	5.5	1.65
Allyl sulfide	C6H10S	114.21	6.4	0.83
Allyl methyl disulfide	C4H8S2	120.24	10.0	4.58
1,3-Dithiane	C4H8S2	120.24	10.2	1.12
Dimethyl trisulfide	C2H6S4	126.26	13.0	0.82
Tetrahydro-2H-1,4,6-oxodiazocine ring	C5H10N2OS	146.00	15.5	5.52
Diallyl disulfide	C8 H12O4	172.18	16.1	17.32
2-Vinyl-1,3-dithiane	C6H10S2	146.00	16.3	8.85
*N*,*N*-Dimethylthiourea	C3H8N2S	104.17	17.2	8.00
Ethylthiourea	C3H8N2OS	120.17	17.7	0.12
2-Ethyltetrahydrothiophene	C6H12S	116.22	19.2	13.24
(Methylthio)acetonitrile	C3H5NS	87.14	19.3	1.40
H1-Propyl-1-(fringyl)-butane	C7H1S	118.00	20.8	1.35
5-Methyl-1,2,4-triazole-3-decanol	C3H5N3S	115.16	23.0	0.50
3-Vinyl-3,4-dihydro-1,2-dithiazolone	C6H8S2	144.26	23.2	3.88
2-Ethylene-1,3-dithiane	C6H10S2	146.00	23.9	0.67
3-Vinyl-3,4-dihydro-1,2-dithiane	C6H8S2	144.26	24.8	17.42
3,5-Diethyl-1,2,4-tritetrahydrothiophene	C6H12S3	180.35	25.2	0.54
1,3,5-Trithiane	C3H6S3	138.27	25.8	0.29
(2-Arylthio)-acetonitrile	C5H7NS	113.00	25.9	0.19
Diallyl trisulfide	C6H10S3	178.34	26.1	2.98
1,3,5-Trithiane	C3H6S3	138.27	27.3	0.76
2-*n*-Propylthiophene	C7H10S	126.22	27.6	0.85
(2-Arylthio)-acetonitrile	C5H7NS	113.00	28.0	0.45
1,3,5-Trithiane	C3H6S3	138.27	29.9	0.36
1,2-Dithiocyclopentane	C3H6S2	106.21	30.7	0.39
2-Thiophene	C4H3NO_2_S	129.14	34.5	1.34

### The Antioxidant Properties of Black Garlic

#### Clearance Rate of DPPH-Free Radicals of Black Garlic

**FIGURE 2:**
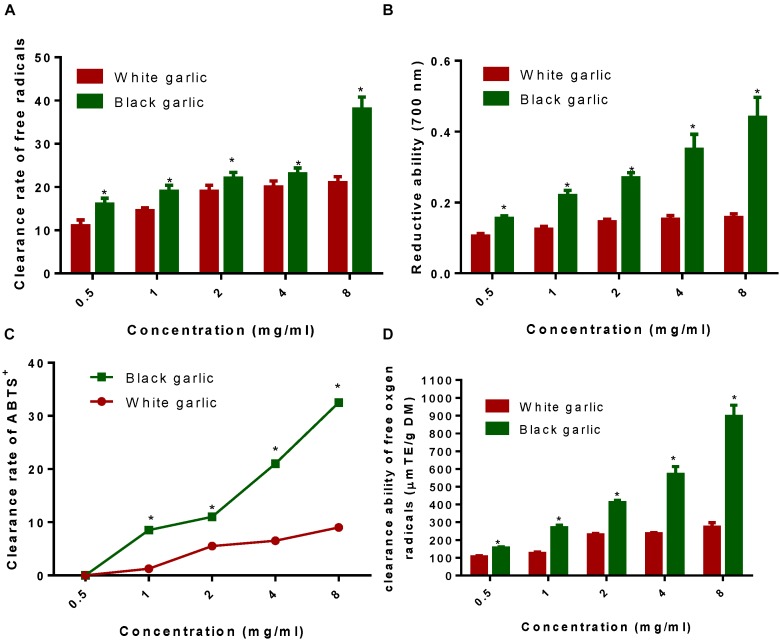
The antioxidant properties of BG. **(A)** Clearance rate of DPPH-free radicals of BG. **(B)** Reduction ability of BG. **(C)** Clearance rate of ABTS radicals of BG. **(D)** Clearance rate of oxygen-free radicals of BG. ^∗^Stands for *P* < 0.05 vs. white garlic.

2,2-Diphenyl-1-picrylhydrazyl is a free radical with a single electron, stable nitrogen center, and is widely used for evaluating the antioxidant properties of plant extracts. When a free radical scavenger is present, the DPPH radical accepts an electron or hydrogen atom to form a stable compound that changes its solution from deep purple to pale yellow, and the degree of discoloration is quantitatively related to the number of electrons. In the present study, the absorbance value was measured with a microplate reader. As shown in Figure 2A, BG had DPPH-free radical scavenging ability, and as the concentration increasing, the DPPH scavenging ability increased. This may be due to the increase of polyphenols content in BG processing, and enhanced antioxidant capacity and free radical scavenging capacity.

#### Reduction Ability of Black Garlic

Reducing ability is a commonly used method for evaluating antioxidant activity. According to the reduction effect of the sample, electrons are scavenged free radicals. As shown in Figure 2B, the reducing ability of BG at the same concentration was significantly higher than that of raw garlic. In this experiment, the total phenol content of raw garlic was 0.49 mg GAeq/g (equivalents of gallic acid per gram of the sample), and the total phenol content of BG reached 2.60 mg GAeq/g, which was five times as much as raw garlic. The results suggested that BG had stronger reducing ability than raw garlic.

#### Clearance Rate of ABTS Radicals of Black Garlic

As shown in Figure 2C, ABTS clearance rate of BG was significantly higher than raw garlic. There was a significant difference in increase clearance rate. At 0.05 mg/mL, raw garlic and BG extracts had no scavenging effect on ABTS. With the increase of concentration, the clearance rate of ABTS was increased. Clearance rate of BG was stronger than that of raw garlic. Generally, the ABTS-free radical scavenging capacity of the BG extract was better than that of raw garlic. On the one hand, it might be related to the content of polyphenols.

#### Clearance Rate of Oxygen-Free Radicals of Black Garlic

The antioxidant principle of ORAC refers to the fact that free radicals can destroy the fluorescent probe and change the fluorescence intensity. The magnitude of its change reflects the degree of free radical damage. Antioxidants can inhibit the change of fluorescence caused by free radicals, and the degree of inhibition can reflect the magnitude of their antioxidant capacity against free radicals. As shown in Figure 2D, both raw garlic and BG could scavenge oxygen-free radicals, but BG had stronger scavenging ability than raw garlic. Raw garlic had an oxygen radical absorption capacity of 324.43 μmol TE/gDM. The absorption capacity of BG reached 984.56 μmol TE/gDM, and the difference was significant (*P* < 0.05). This was consistent with the results of DPPH and Fe^3+^ reducing ability, which was due to the fact that the polyphenol content in BG was significantly higher than that of raw garlic.

### Clinical Characteristics

There was no significant statistical differences for clinical characteristics of CHF patients between BG and CG groups, including gender distribution, body mass index (BMI), age, diastolic blood pressure (DBP), and systolic blood pressure (SBP) (Table 3, *P* > 0.05). The cases for taking ACE-In, ARBS, beta-blockers, diuretics, and performing CRT and ICD therapies were comparable between two groups (*P* > 0.05, Table 3).

**Table 3:** Clinical characteristics between BG and placebo groups.

Parameters	BG	Placebo	χ^2^-and *t*-value	*P*-value
Gender (male/female)	32/28	34/26	0.135	0.714
Age (year)	39.89 ± 13.26	40.23 ± 12.78	-1.303	0.179
SBP (mm Hg)	126.21 ± 11.52	130.54 ± 12.76	-1.684	0.086
DBP (mm Hg)	87.23 ± 7.16	86.53 ± 7.48	-1.296	0.158
BMI	24.91 ± 1.74	24.52 ± 1.48	-1.563	0.098
TC (mmol/L)	5.52 ± 0.64	5.72 ± 0.87	-0.618	0.274
TG (mmol/L)	2.24 ± 0.81	2.32 ± 0.94	-2.158	0.109
LDL-C (mmol/L)	2.01 ± 0.64	2.35 ± 0.83	-1.864	0.187
HDL-C (mmol/L)	1.84 ± 0.46	1.65 ± 0.32	-2.619	0.074
Cr (μmol/L)	85.24 ± 13.58	87.04 ± 14.51	-1.244	0.136
HbA1C (%)	8.42 ± 0.73	8.72 ± 0.86	-0.654	0.246
ACE-In	6	5	3.07	0.38
ARBS	3	7		
Beta-blockers	4	3		
Diuretics	8	4		
CRT	6	9	0.68	0.41
ICD	2	4	0.18	0.68

*Chi-square test and t-test were used to compare the significant difference between COG and CG groups. BMI, body mass index; ACE-In, angiotensin-converting enzyme; ARBS, angiotensin receptor blockers; CRT, cardiac resynchronization therapy; ICD, implantable cardioverter defibrillator. All data were presented as mean value ± SD. There were significantly statistical differences between two groups if P < 0.05.*

### Therapeutic Results of Black Garlic

There was no significant difference (*P* > 0.05) in the mean values of BUN and before BG therapy (*P* < 0.05, Table 4). After therapy, the values of mean BUN and serum creatinine were significantly reduced in both groups (*P* < 0.05, Table 4). Meanwhile, the values of mean BUN and serum creatinine were significantly reduced when compared with placebo groups (*P* < 0.05, Table 4).

**Table 4:** The therapeutic results of BG.

		Before treatment	After treatment	*t*-values	*P*-values
Blood urea nitrogen (mg/dL)	BG	19.03 ± 6.85	15.23 ± 5.24	6.41	0.01^a^
	Placebo	18.71 ± 6.34	17.05 ± 5.98	2.16	0.03^a^
	*t*-values	0.37	3.28		
	*P*-values	0.51	0.02		
Serum creatinine (mg/dL)	BG	1.41 ± 0.32	1.04 ± 0.27	8.65	0.01^a^
	Placebo	1.36 ± 0.29	1.20 ± 0.19	2.39	0.04^a^
	*t*-values	0.24	4.31		
	*P*-values	0.63	0.02		

*^a^Stands for P < 0.05 vs. before treatment.*

### Comparison of Two Sets of Lee Scores

Before BG treatment, the statistical difference for Lee scores was insignificant between BG and CG groups (Table 5, *P* > 0.05). After BG consumption, the statistical differences for Lee scores were significant in both BG and CG groups when compared with before treatments (Table 5, *P* < 0.05). BG reduced more Lee scores than CG (Table 5, *P* < 0.05).

**Table 5:** The comparison of Lee scores between two groups.

	Before treatment	After treatment	*t*-values	*P*-values
BG	4.02 ± 1.81	1.18 ± 0.40	11.38	0.01^a^
Placebo	4.23 ± 1.76	2.19 ± 0.73	9.25	0.01^a^
*t*-values	0.25	4.12		
*P*-values	0.68	0.02^b^		

*n = 60 for each group. ^a^P < 0.05 vs. before treatment and ^b^P < 0.05 vs. a placebo group.*

### The QOL Scores

Before BG consumption, the statistical difference for the QOL scores was insignificant between BG and CG groups (Table 6, *P* > 0.05). After 6-month BG consumption, the statistical differences for the QOL scores were significant in both BG and CG groups (Table 6, *P* < 0.05). BG increased more QOL scores than CG (Table 6, *P* < 0.05).

**Table 6:** The comparison of QOL between two groups.

	Before treatment	After treatment	*t*-values	*P*-values
BG	43.68 ± 3.38	21.76 ± 4.18	23.40	0.01^a^
Placebo	42.55 ± 3.05	29.39 ± 4.16	14.85	0.01^a^
*t*-values	0.34	2.19		
*P*-values	0.78	0.02^b^		

*n = 60 for each group. ^a^P < 0.05 vs. before treatment and ^b^P < 0.05 vs. a placebo group.*

### Comparison of 6-Min Walk Distance Between Two Groups

Before BG consumption, the statistical difference for 6-min walk distance was insignificant between BG and CG groups (Table 7, *P* > 0.05). After 6-month BG consumption, the statistical differences for 6-min walk distance were significant in both BG and CG groups (Table 7, *P* < 0.05). Meanwhile, BG increased more 6-min walk distance than CG (Table 7, *P* < 0.05).

**Table 7:** The comparison of 6-min walk distance between two groups (m).

	Before treatment	After treatment	*t*-values	*P*-values
BG	356.22 ± 41.93	426.16 ± 29.96	8.47	0.01^a^
Placebo	348.32 ± 36.79	372.83 ± 28.16	2.12	0.08
*t*-values	0.298	2.41		
*P*-values	0.649	0.02^b^		

*n = 60 for each group. ^a^P < 0.05 vs. before treatment and ^b^P < 0.05 vs. a placebo group.*

### Comparison of Nt-proBNP Concentration Between Two Groups

Before BG consumption, the statistical difference for Nt-proBNP concentration was insignificant between BG and CG groups (Table 8, *P* > 0.05). After 6-month BG consumption, the statistical differences for Nt-proBNP concentration were significant in both BG and CG groups when compared with before treatments (Table 8, *P* < 0.05). Meanwhile, the concentration of Nt-proBNP was reduced by 18.47 ± 2.69% in the BG group when compared with the CG group (Table 8, *P* < 0.05).

**Table 8:** The comparison of Nt-proBNP between two groups (pg/mL).

	Before treatment	After treatment	*t*-values	*P*-values
BG	1895.12 ± 249.34	1291.64 ± 207.04	5.94	0.01^a^
Placebo	1861.63 ± 257.13	1536.54 ± 216.32	2.65	0.04^a^
*t*-values	0.10	1.95		
*P*-values	0.87	0.03^b^		

*n = 60 for each group. ^a^P < 0.05 vs. before treatment and ^b^P < 0.05 vs. a placebo group.*

### Comparison of LVEF Volume Between Two Groups

Before BG consumption, the statistical difference for LVEF volume was insignificant between BG and CG groups (Table 9, *P* > 0.05). After 6-month BG consumption, the statistical differences for LVEF volume were significant in both BG and CG groups when compared with before treatments (Table 9, *P* < 0.05). Meanwhile, the values of LVEF volume were improved by 14.29 ± 4.38% in the BG group when compared with the CG group (Table 9, *P* < 0.05).

**Table 9:** The comparison of LVEF between two groups.

	Before treatment	After treatment	*t*-values	*P*-values
BG	29.36 ± 9.34	36.82 ± 10.43	5.03	0.01^a^
Placebo	28.24 ± 8.15	32.73 ± 10.21	3.10	0.01^a^
*t*-values	0.98	2.14		
*P*-values	0.36	0.03^b^		

*n = 60 for each group. ^a^P < 0.05 vs. before treatment and ^b^P < 0.05 vs. a placebo group. LVEF, left-ventricular ejection fraction.*

### Adverse Reaction

No obvious adverse reactions occurred during the whole experiment, suggesting that the drug is safe in treatment.

### Circulating Antioxidant Levels

**FIGURE 3:**
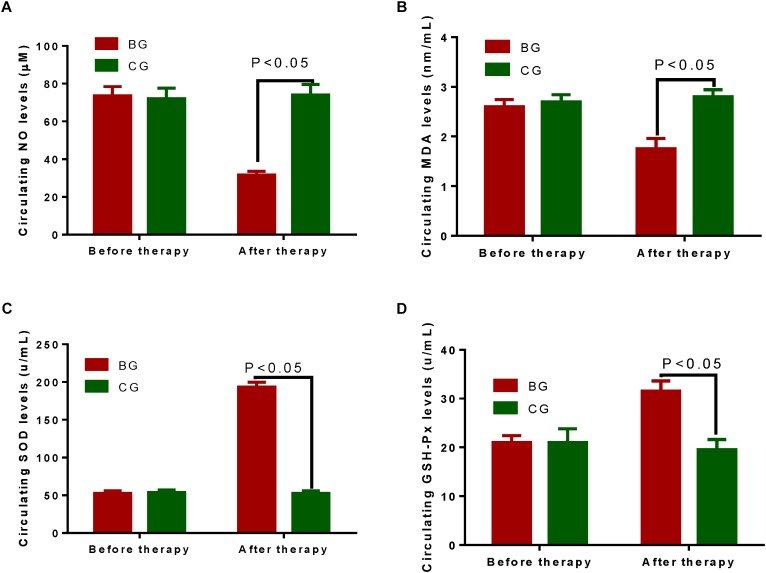
Circulating antioxidant levels between two groups. **(A)** Circulating levels of NO between two groups. **(B)** Circulating levels of MDA between two groups. **(C)** Circulating levels of SOD between two groups. **(D)** Circulating levels of GSH-PX between two groups. The statistical difference was significant if *P* < 0.05.

Circulating antioxidant levels were investigated between two groups. The statistical difference for the biomarkers was insignificant between two groups before therapy (Figure 3, *P* > 0.05). After therapy, circulating levels of NO (Figure 3A) and MDA (Figure 3B) were lower in BG than in CG group while the circulating levels of SOD (Figure 3C) and GSH-PX (Figure 3D) were higher in BG than in CG group (*P* < 0.05).

## Discussion

Recent studies have shown that BNP has a high reliability in the diagnosis of CHF and provides an important method for heart failure diagnosis (Booth et al., 2014). BNP has been listed as one of the diagnostic criteria for heart failure by the American College of Cardiology (ACC) and the European Society of Cardiology (ESC) (Fonseca et al., 2004; Emdin et al., 2007). According to an earlier report, the measuring limits of BNP, proBNP, and NT-proBNP were 0.4, 3, and 10 pg/mL, respectively (Seferian et al., 2007). The study showed that BNP concentrations were elevated, and cardiac function indicators were highly expressed. There was a significant correlation between cardiac dysfunction and NT-proBNP concentration, suggesting that BNP concentration measurement can be used as an effective method to screen patients with CHF, and it will be effective in the early diagnosis of CHF.

The concentration of plasma BNP was positively correlated with NYHA classification. The worse of heart function, the higher the severity of heart failure and the higher concentration of plasma BNP. In the present experiment, the most patients with cardiac function III and IV and Nt-proBNP were significantly decreased after receiving BG (Table 8). This study showed that the plasma BNP concentration value could be used as a reliable indicator to judge the severity of heart failure, and it was easy to operate.

The determination of plasma BNP concentration has been considered as a powerful indicator in prognostic evaluation of CHF risk. High-level plasma BNP, especially before treatment, showed a poor prognosis. Comparatively, the BNP concentration decreased (average 215 pg/mL) in patients who did not have a cardiac endpoint (Cheng et al., 2001). In addition, BNP concentration was reported to be an independent risk predictor of death in CHF patients (Richards et al., 2001). The heart failure patients with BNP >700 pg/mL and the 120-day mortality rate and readmission rate were 80%. In contrast, the patients with BNP values < 350 pg/mL had mortality and the readmission rate was <10% (Logeart et al., 2004). The level of BNP not only reflects the severity of heart failure, but also is an effective prognostic indicator of heart failure. The results of this study showed that the Nt-proBNP levels were significantly higher in the patients with heart failure. BNP levels were powerful indicators for the diagnosis of heart failure. The results also showed that BG consumption resulted in significant reduction in the level of Nt-proBNP compared with that before the treatment and CG group (*P* < 0.05), suggesting that BG consumption can reduce plasma N-terminal BNP levels in heart failure patients, antagonize neuroendocrine activation, and improve cardiac function. Meanwhile, therapeutic results of BG were approved by reducing the values of mean BUN and serum creatinine when compared with placebo groups (*P* < 0.05, Table 4).

Hambrecht study found that rehabilitation exercise on CHF lowered the resting heart rate during submaximal exercise, and increased maximal oxygen uptake, exercise tolerance and anaerobic threshold, physical activity, and QOL (Hambrecht et al., 2000). CHF can cause skeletal muscle mechanoreceptor activation leading to increased ventilation and chest tightness sensation, as well as fatigue and sympathetic activation, and at least one-fourth of CHF patients are caused by skeletal muscle abnormalities (Rogers, 2001). Thus, all patients received 30-min walking exercise daily.

The results of this study also showed that the levels of LVEF in both BG and CG groups were improved when compared with before treatment (*P* < 0.05). The scores of the diagnosis of heart failure were decreased (*P* < 0.05). Medicine combined with BG effectively improved heart failure patients with lower LVEF (*P* < 0.05).

In this study, the 6-min walk test was used as a performance evaluation indicator for CHF patients. The 6-min walk test was used as an objective indicator to evaluate the activity, physical fitness, and drug intervention effects of patients with heart failure. The 6-min walking experiment has been studied in more depth and widely used (Brehm et al., 2017; Fakhro et al., 2017; Omar and Guglin, 2017).

The results of this study showed that the QOL scores were improved after walking exercise in both groups compared with before the excise (*P* < 0.05); and after BG treatment, the improvement was better in the BG group than that in CG group (*P* < 0.05); suggesting that BG will be better than CG for improving the living ability of patients with heart failure.

**FIGURE 4:**
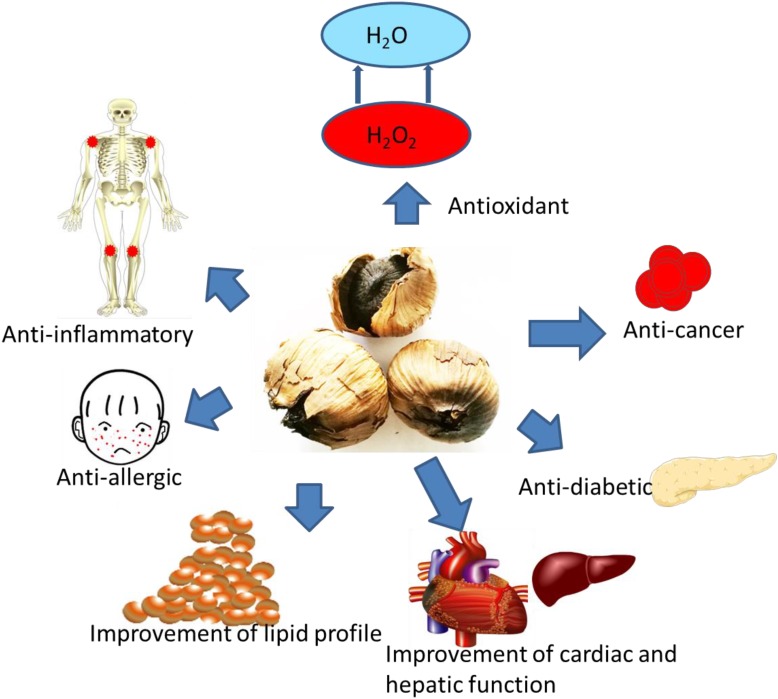
Health-promoting properties of BG.

After therapy, circulating levels of NO (Figure 3A) and MDA (Figure 3B) were lower in BG than in CG group while the circulating levels of SOD (Figure 3C) and GSH-PX (Figure 3D) were higher in BG than in CG group (*P* < 0.05). The results suggest that BG improves the antioxidant properties of CHD patients. Antioxidant levels had positive relation with QOL and LVEF values, and negative relation with Nt-proBNP values. The improvement of QOL has been demonstrated in the patients who have received antioxidant therapy (Shah et al., 2010). Nt-proBNP is an important biomarker for reflecting total oxidized stress in the patients with acute myocardial infarction (Kasap et al., 2007). On the other hand, antioxidant therapy can improve LVEF values (de Lorgeril et al., 2001). Thus, BG may improve the symptoms of CHD patients by increasing antioxidant activities. Black garlic has many health-promoting properties (Figure 4). Considering the short time of the present study, the small sample size and other influencing factors, further work is highly demanded to confirm the present result.

## Conclusion

Black garlic improved blood circulation in the treatment of CHD patients. BG combined with conventional treatment improved the LVEF and heart function, and reduced the heart failure diagnosis. BG treatment improved the QOL, the patient’s actual living ability, and QOL. BG consumption increased the distance of 6-min walking test in CHD patients and showed obvious advantages in the recovery of physical function. BG combined with medicine treatment reduced plasma N-terminal pro-body BNP levels and antagonized neuroendocrine activation in CHD patients.

## Author Contributions

JL and CW designed the study. JL, GZ, XC, and CW performed the experiments. GZ analyzed the data. CW wrote the manuscript. JL and XC revised and corrected the manuscript. All authors read and approved the final manuscript.

## Conflict of Interest Statement

The authors declare that the research was conducted in the absence of any commercial or financial relationships that could be construed as a potential conflict of interest.

## Footnotes

1 http://www.chictr.org.cn/showprojen.aspx?proj=30330
